# Supplementary material for: Extensive allele-specific translational regulation in hybrid mice
Source: Mol Syst Biol. 2015 Aug 7;11(8):825. doi: 10.15252/msb.156240 (PMC4562498; doi:10.15252/msb.156240)
Supplement: Supplementary file 1 — Expanded View Figures PDF [file msb0011-0825-sd1.pdf]

Expanded View Figures

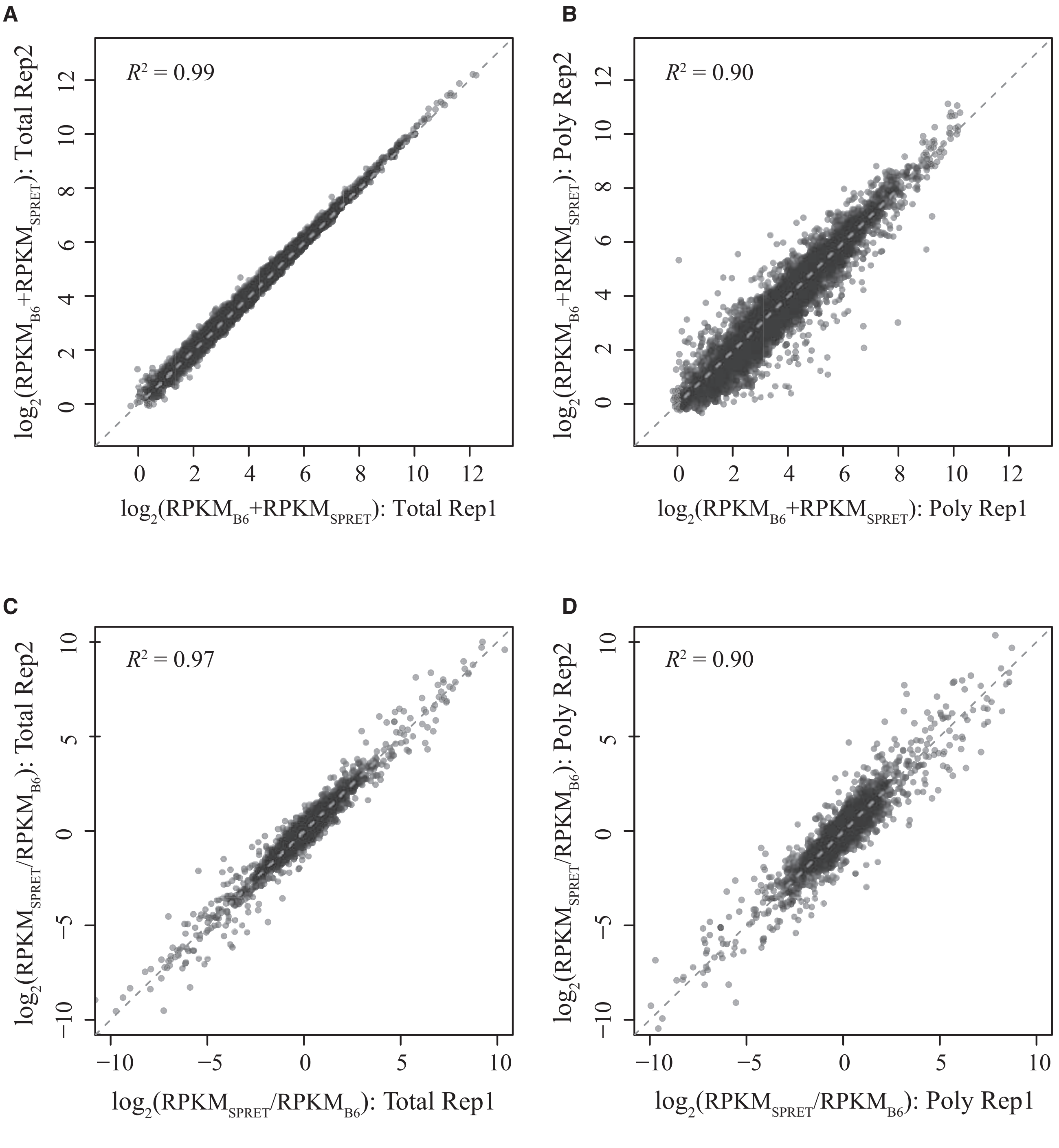

Figure EV1. Reproducibility of mRNA sequencing and polysome profiling data.

A Scatterplot comparing the abundance of cellular mRNA (sum of both alleles) (log2-transformed RPKM values for total mRNA) between two biological replicates. Each dot represents one gene.

B Scatterplot comparing the abundance of polysome-associated mRNA (sum of both alleles) (log2-transformed RPKM values for poly-mRNA) between two biological replicates. Each dot represents one gene.

C Scatterplot comparing the log2-transformed allelic ratio of cellular mRNA abundance between two biological replicates. Each dot represents one gene.

D Scatterplot comparing the log2-transformed allelic ratio of polysome-associated mRNA abundance between two biological replicates. Each dot represents one gene.

© 2015 The Authors

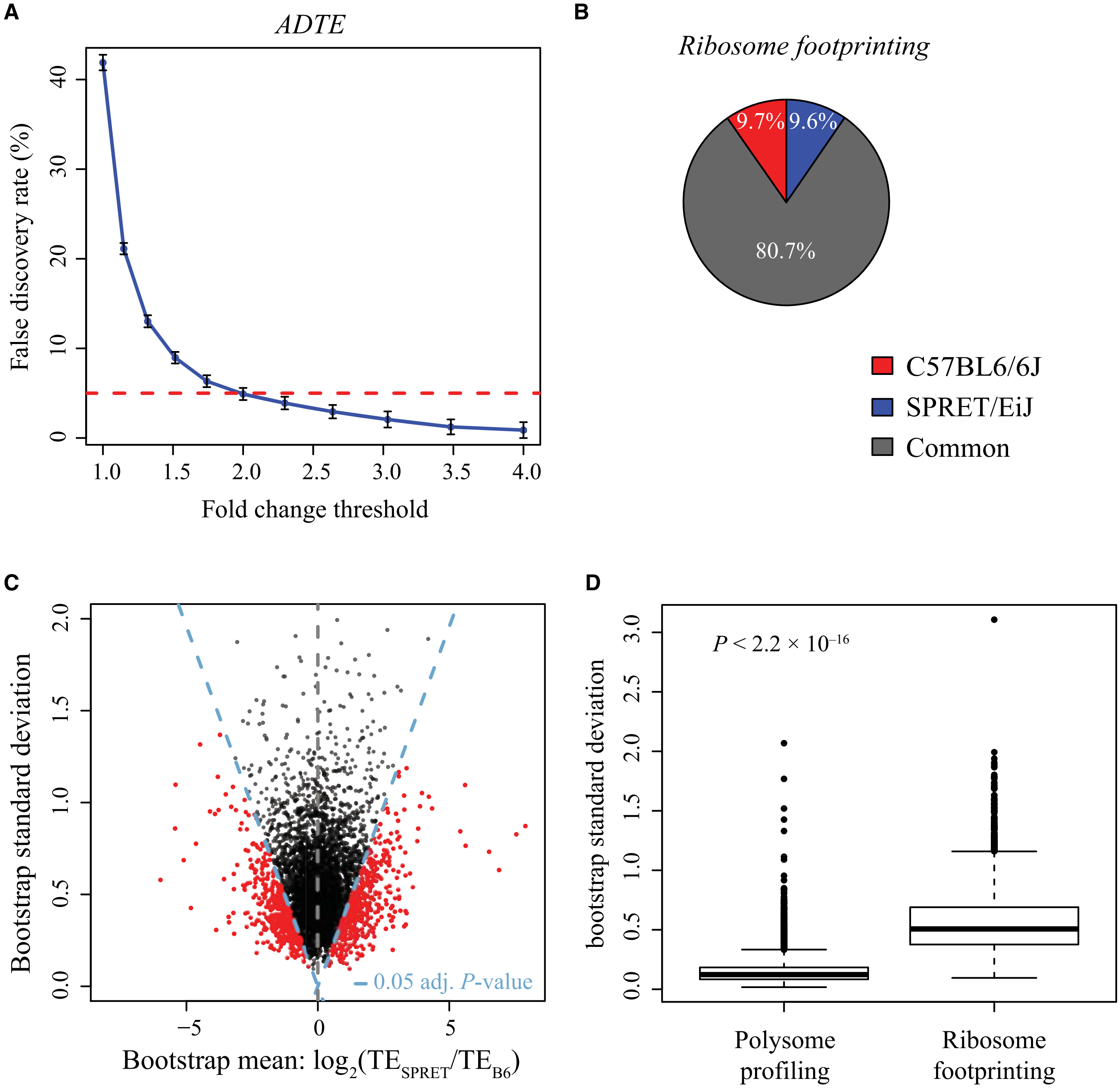

Figure EV2. ADTE estimation based on polysome profiling and ribosome footprinting data.

A False discovery rate (FDR, shown in *y*-axis) was plotted against different allelic fold change cut-off (*x*-axis) in identifying genes with significant ADTE. To determine the FDR based on our biological replicates, we applied a similar permutation strategy as that used in Sterne-Weiler *et al* (2013). In short, gene labels were shuffled for 100 times in both replicates, and in each of the 100 shuffled sets, we estimated false discovery findings by counting the number of genes in both replicates meeting the fold change (FC) requirement ($|FC| > x$) and bootstrapping significance requirement (adjusted *P*-value < 0.05), and biased towards the same allele, denoted as FP (x). Then, the FDR in each set for each value of x was estimated as FP(x) divided by the number of real genes passing the same criteria. Error bars represent s.d.

B The percentage of uniquely mapped RFP reads that could be unambiguously assigned to C57BL/6J (red) and SPRET/EiJ (blue) alleles or assigned to the two alleles with equal probability (common, grey). Only 19.3% RFP reads could be assigned unambiguously with allelic origin.

C Scatterplot showing the bootstrap means (*x*-axis) and standard deviations (*y*-axis) in estimating ADTE for the 4,511 genes containing at least five coding SNPs supported with sufficient allelic RFP reads. Dashed blue lines indicate the adjusted *P*-value of 0.05. Genes with significant ADTE (adjusted *P*-value < 0.05) are depicted in red.

D The distribution of bootstrap standard deviation of polysome profiling (left) and ribosome footprinting data (right). The uncertainty in ADTE estimation (i.e. bootstrapping standard deviation) based on polysome profiling was significantly lower than that based on ribosome footprinting ($P < 2.2 \times 10^{-16}$, Mann–Whitney *U*-test).

© 2015 The Authors

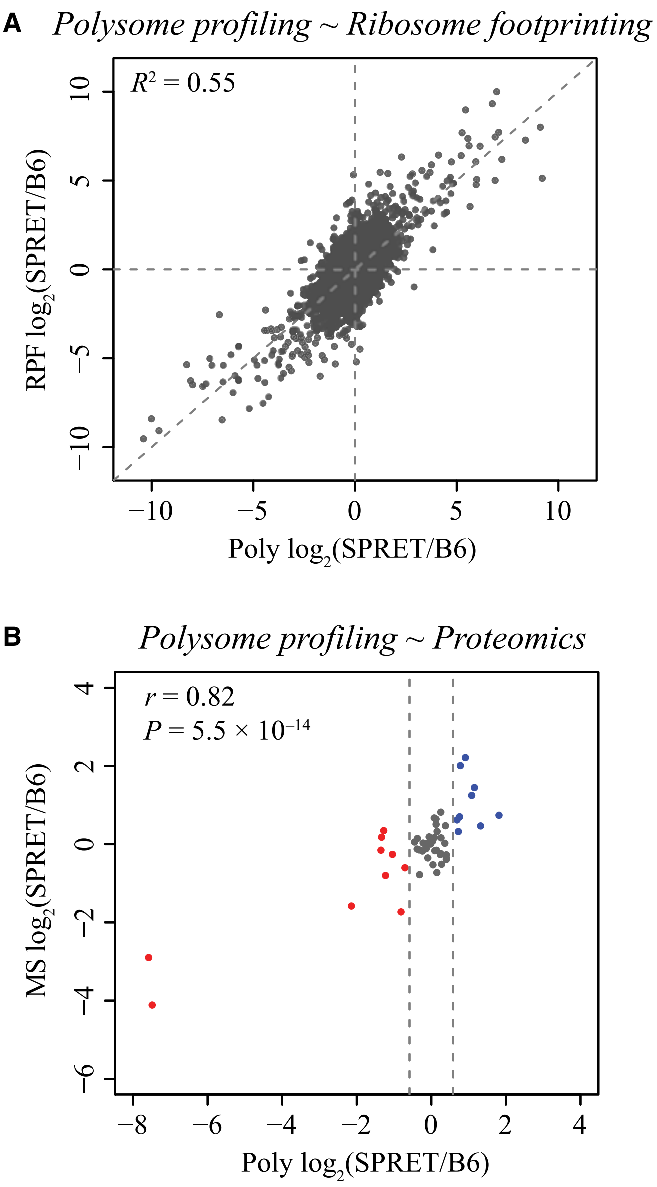

Figure EV3. Allelic divergence in translational status estimated based on polysome profiling data correlated with that based on ribosome footprinting data and proteomics data.

A Scatterplot comparing the log2-transformed allelic ratio of polysome-associated mRNA abundance (*x*-axis) versus that of the abundance of ribosome-protected fragments (*y*-axis). Each dot represents one gene. The R^2 of 0.55 indicates significant correlation between polysome profiling and ribosome footprinting data in quantifying allelic biases in translational status.

B Scatterplot comparing the log2-transformed allelic ratio of polysome-associated mRNA abundance (*x*-axis) versus that of protein abundance using MS-based proteomics data (*y*-axis). Colour labels are the same as those used in Fig 2D.

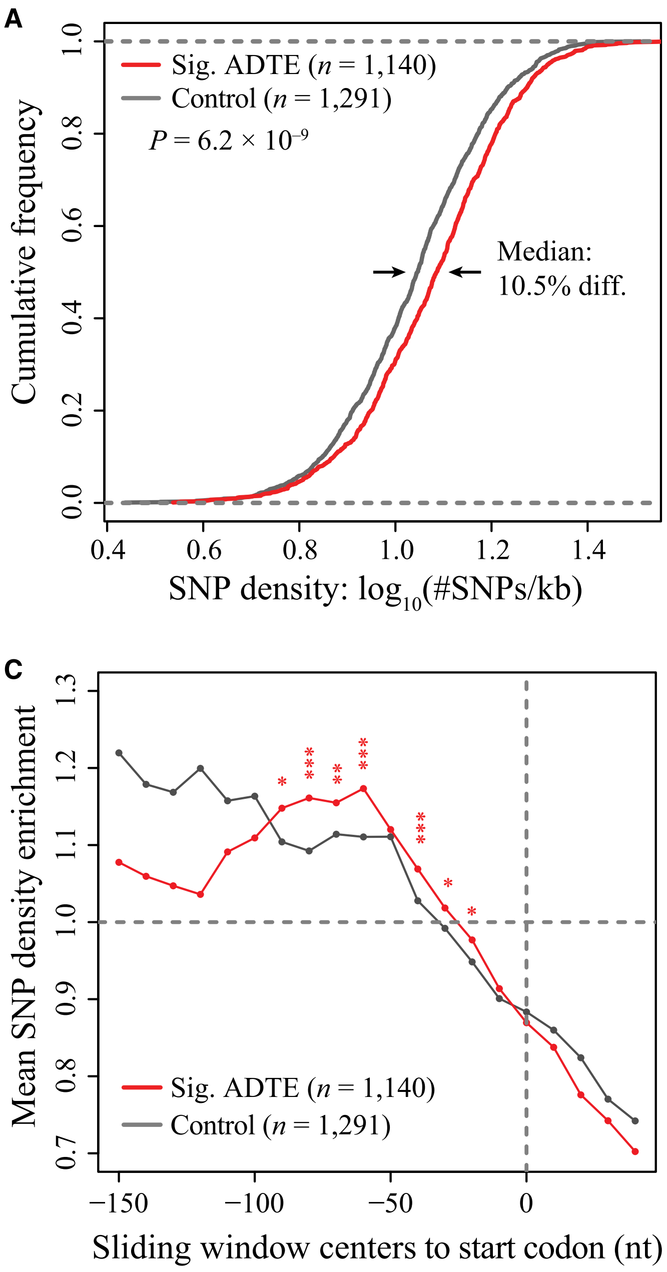

Figure EV4. Sequence features that were correlated with ADTE, based on the polysome profiling data with ADTE cut-off = 1.5.
A–C Panels are the same as Fig 3A–C, but with ADTE cut-off = 1.5. Significant differences were indicated by asterisks (*BH-adjusted $P < 0.05$, **BH-adjusted $P < 0.01$, ***BH-adjusted $P < 0.001$).

 © 2015 The Authors

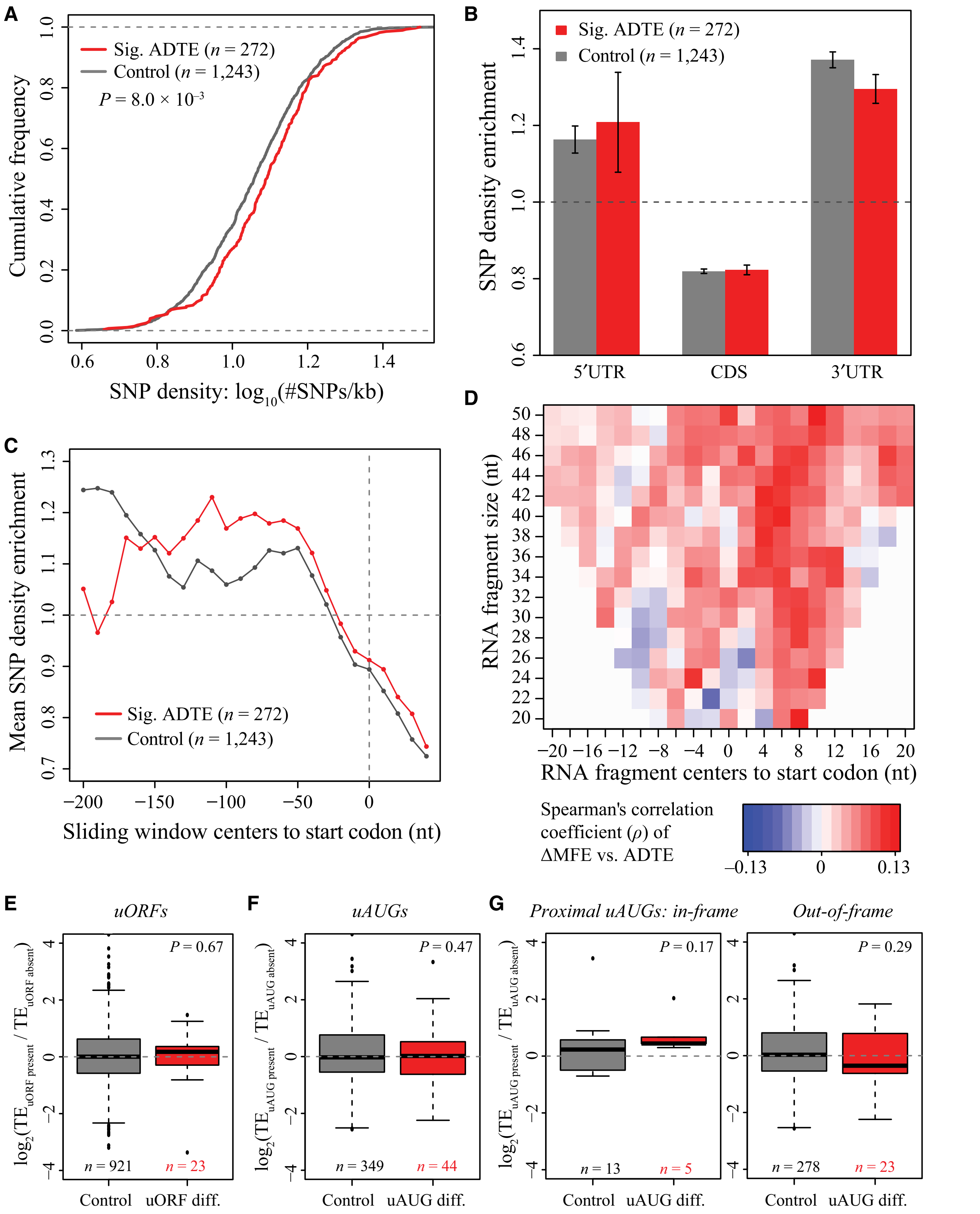

Figure EV5. Sequence features that were correlated with ADTE, based on the ribosome footprinting data.

A–G Panels are the same as Fig 3A–G, but based on ribosome footprinting data.

© 2015 The Authors

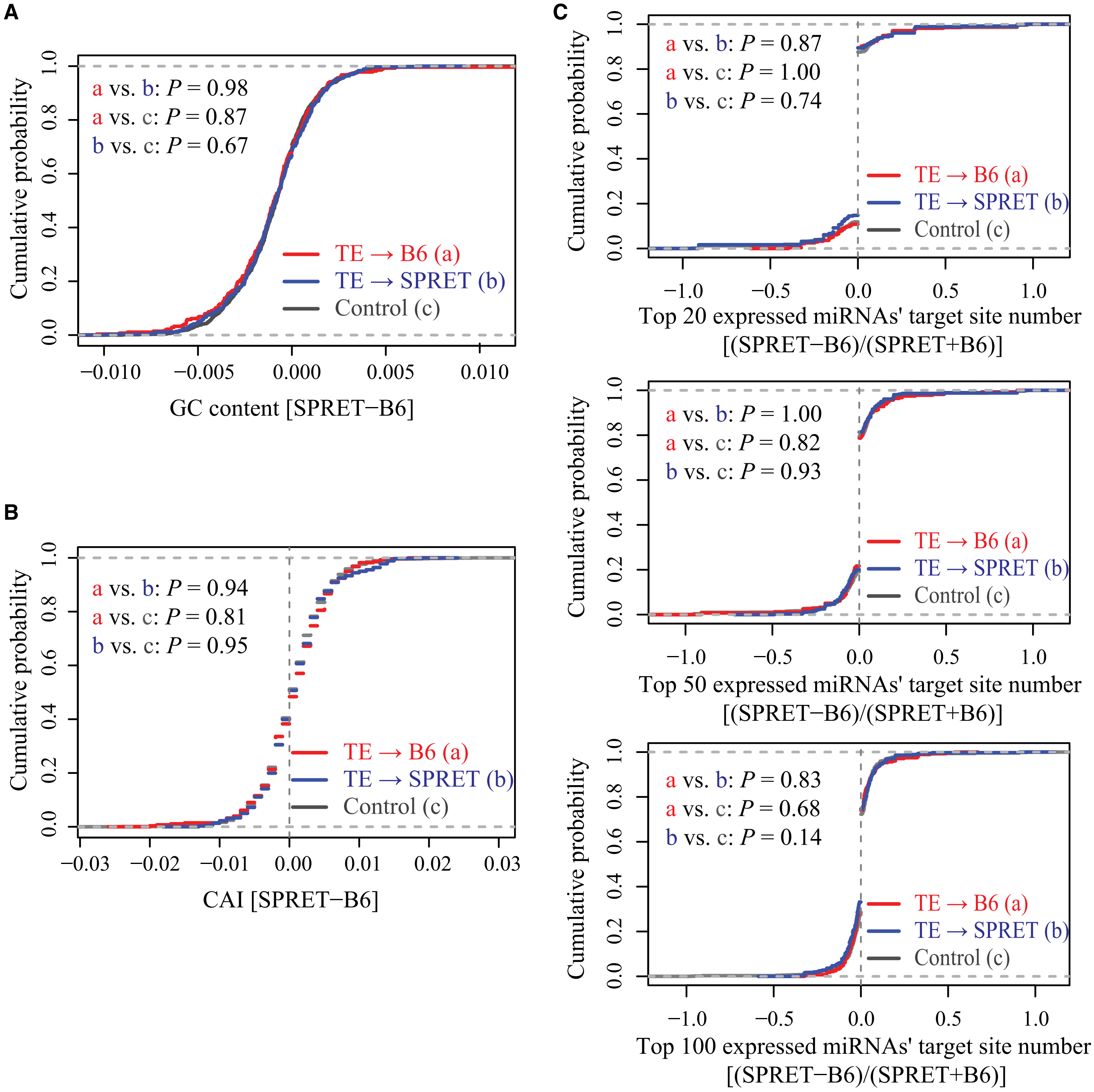

Figure EV6. Sequence features that did not affect TE.

A The allelic differences in GC content had no significant correlation with observed ADTE ($P > 0.05$ for all pairwise comparisons, Kolmogorov–Smirnov test). Genes examined for ADTE were separated into three sets: TE significantly bias towards C57BL/6J (red), towards SPRET/EiJ (blue) and no bias (control grey) (see Materials and Methods). The cumulative distribution function (CDF) of allelic difference in GC content was plotted for the three gene sets separately.

B The allelic differences in codon adaptation index (CAI) had no significant correlation with observed ADTE ($P > 0.05$ for all pairwise comparisons, Kolmogorov–Smirnov test). The cumulative distribution function (CDF) of allelic difference in CAI was plotted for the three gene sets (see above) separately.

C The allelic differences in the number of miRNA binding sites had no significant correlation with observed ADTE ($P > 0.05$ for all pairwise comparisons, Kolmogorov–Smirnov test). The cumulative distribution function (CDF) of allelic difference in the binding sites for the top 20 (top), 50 (middle) and 100 (bottom) expressed miRNAs was plotted for the three gene sets (see above) separately.

 © 2015 The Authors

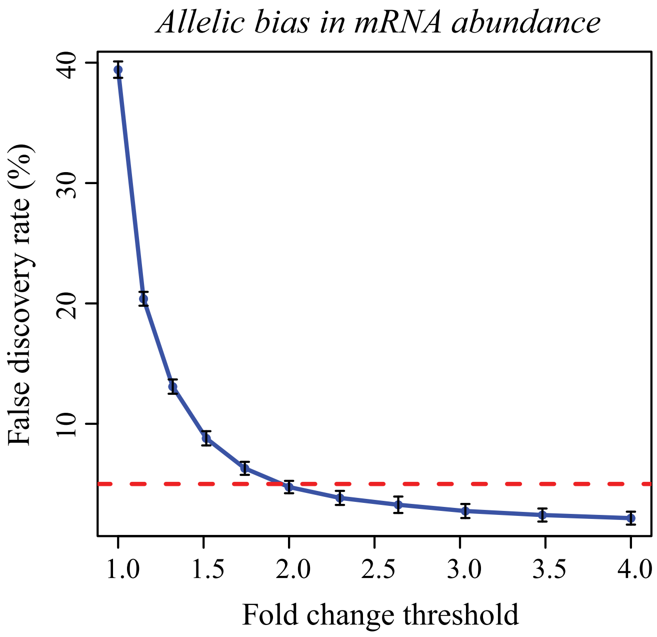

Figure EV7. False discovery rate (FDR) in identifying genes with significant allelic bias in mRNA abundance.
FDR was plotted against different allelic fold change cut-off in identifying genes with significant allelic bias in mRNA abundance. To determine the FDR based on our biological replicates, we applied the same permutation procedure as that in the ADTE analysis (see Fig EV2A). Error bars represents s.d.

© 2015 The Authors

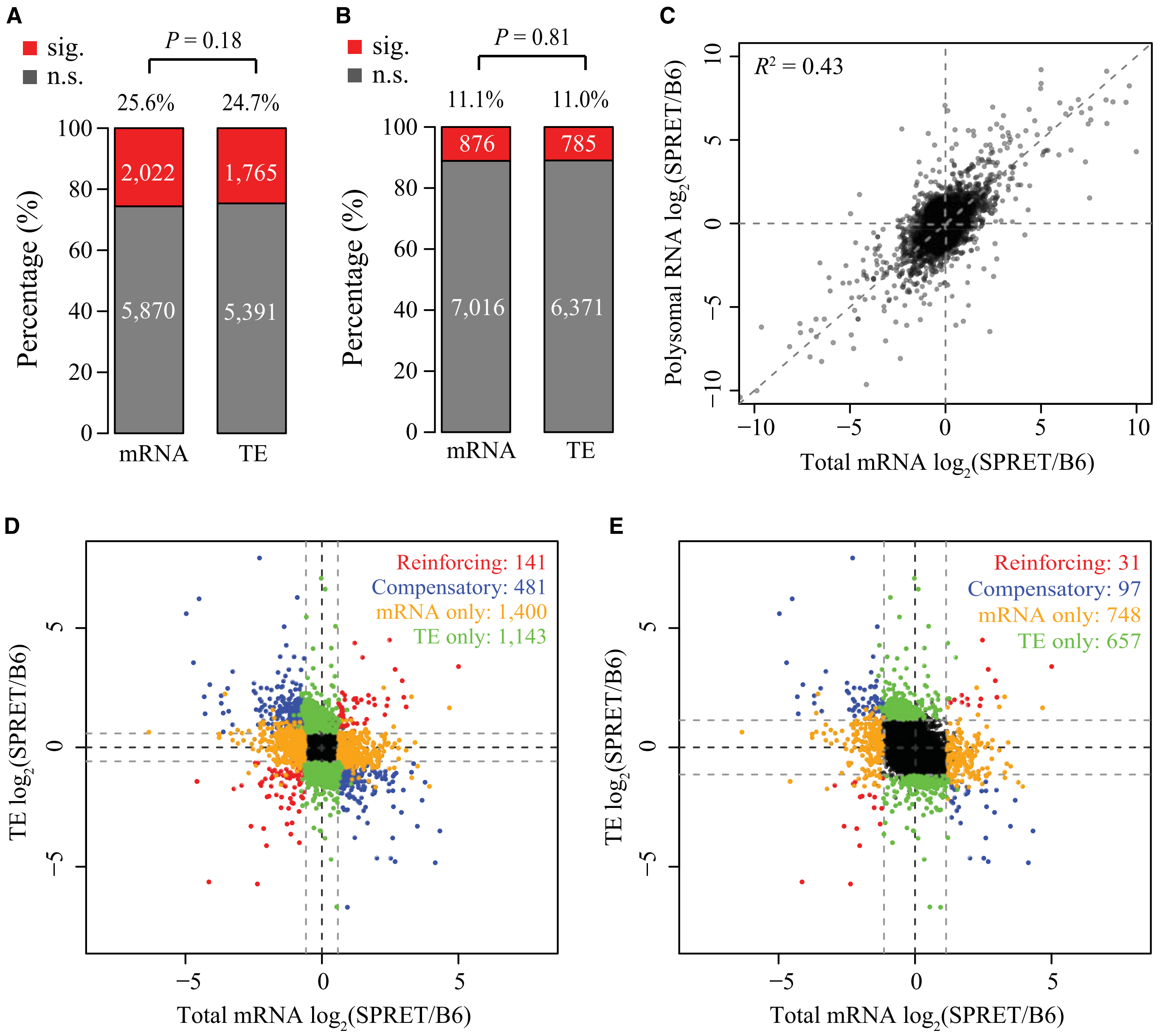

Figure EV8. Allelic regulation at both transcriptional and translational levels with different significance cut-offs.

A, B Panels are the same as Fig 4A, but with allelic fold change cut-off = 1.5 (A) or 2.2 (B).

C Scatterplot comparing the log2-transformed allelic ratio of cellular mRNA abundance (*x*-axis) versus that of polysome-associated mRNA abundance (*y*-axis). Each dot represents one gene. The R^2 of 0.43 indicates that less than half of allelic divergence in polysome-associated RNA abundance could be explained by the allelic divergence in mRNA cellular abundance.

D, E Panels are the same as Fig 4B, but with allelic fold change cut-off = 1.5 (D) or 2.2 (E).

 © 2015 The Authors
